# Personal care products: an emerging threat to the marine bivalve *Ruditapes philippinarum*

**DOI:** 10.1007/s11356-024-32391-1

**Published:** 2024-02-20

**Authors:** Marina G. Pintado-Herrera, Gabriela V. Aguirre-Martínez, Laura M. Martin-Díaz, Julián Blasco, Pablo A. Lara-Martín, Marta Sendra

**Affiliations:** 1 Physical Chemistry Department, University of Cadiz, International Campus of Excellence of the Sea (CEI•MAR), 11510 Cadiz, Spain; 2 Faculty of Health Science, Arturo Prat University, Avenida Arturo Prat 2120, Iquique, Chile; 3 Department of Ecology and Coastal Management, Institute of Marine Sciences of Andalusia (CSIC), Campus Rio S. Pedro, 11510 Puerto Real, Cadiz, Spain; 4 Department of Biotechnology and Food Science, Faculty of Sciences, University of Burgos, Plaza Misael Bañuelos, 09001 Burgos, Spain; 5 International Research Center in Critical Raw Materials-ICCRAM, University of Burgos, Plaza Misael Bañuelos S/N, 09001 Burgos, Spain

**Keywords:** Personal care product, Bioconcentration, Clam, PCP post-exposure, Effects, Biomarkers

## Abstract

In the last few decades, there has been a growing interest in understanding the behavior of personal care products (PCPs) in the aquatic environment. In this regard, the aim of this study is to estimate the accumulation and effects of four PCPs within the clam *Ruditapes philippinarum*. The PCPs selected were triclosan, OTNE, benzophenone-3, and octocrylene. A progressive uptake was observed and maximum concentrations in tissues were reached at the end of the exposure phase, up to levels of 0.68 µg g^−1^, 24 µg g^−1^, 0.81 µg g^−1^, and 1.52 µg g^−1^ for OTNE, BP-3, OC, and TCS, respectively. After the PCP post-exposure period, the removal percentages were higher than 65%. The estimated logarithm bioconcentration factor ranged from 3.34 to 2.93, in concordance with the lipophobicity of each substance. No lethal effects were found although significant changes were observed for ethoxyresorufin O-demethylase activity, glutathione S-transferase activity, lipid peroxidation, and DNA damage.

**Supplementary information:**

The online version contains supplementary material available at 10.1007/s11356-024-32391-1.

## Introduction

The rapid socio-economic and industrial development in recent decades has brought a continuous release of chemical substances into water bodies and might have a negative effect on the organisms living in these systems. Despite the high number of organic chemicals detected in aquatic systems, the number of regulated and/or banned substances (Directive 2013/39/EU) is limited. The scientific community has shown greater interest in certain compounds in recent decades, which are not regulated even though they may cause adverse effects on human health and ecosystems. Only some substances are incorporated in the surface water Watch List (European Commission, e.g., 2022/1307/UE) or Toxic Substances Control Act (TSCA, Environmental Protection Agency), which are updated regularly. These unregulated chemicals, known as contaminants of emerging concern (CECs), are widely distributed and their levels in the environment seem to have increased in some places as has been demonstrated by analyzing dated sediment cores (Lara-Martín et al. 2015; Nipen et al. 2022). CECs cover a wide group of natural or synthetic compounds, such as personal care products (PCPs), nanoparticles, microplastics, or pesticides, among others, some of them are considered persistent, bioaccumulative, and toxic substances (PBTs) (Impellitteri et al. 2023; Sauvé and Desrosiers 2014).

Focusing on PCPs, they are a heterogeneous group of chemicals containing UV-filters, fragrances, antibacterial products, insect repellents, or parabens, whose levels are increasing in freshwater (Ebele et al. 2017) and marine environments (Arpin-Pont et al. 2016; Fenni et al. 2022; Nipen et al. 2022; Zicarelli et al. 2022). Their occurrence in the environment is mainly due to effluent discharges since they are not removed during wastewater treatments and recreational activities such as swimming (Labille et al. 2020; Pintado-Herrera et al. 2020). PCPs have been detected in different environmental compartments, such a sediment, water (Cadena-Aizaga et al. 2022; Pemberthy et al. 2020; Pintado-Herrera et al. 2020), and even in organisms (Bayen et al. 2019; Dodder et al. 2014; Pemberthy et al. 2020; Vidal-Liñán et al. 2018). The UV filters octocrylene (OC) and benzophenone-3 (BP-3), the antibacterial triclosan (TCS) and the fragrance OTNE (octahydro-tetramethyl-naphthalenyl-ethanone) are frequently detected in effluent discharges, these PCPs occurred in more than 65% of the samples analyzed, and all of them were found to reach up to a concentration of 7 μg L^−1^ (Biel-Maeso et al. 2019).

According to persistence of chemical substances in tissues, the physical–chemical properties such as n-octanol/water partition coefficient (log *K*_ow_) is crucial since log *K*_ow_ is generally inversely related to water solubility and directly proportional to molecular weight of a substance. These four PCPs, they are considered persistent or pseudopersistent; log *K*_ow_ > 3 (Beyer et al. 2017); furthermore, organisms can accumulate them because they are not fully metabolized or excreted (Dodder et al. 2014) and/or biotransformed (Bonnefille et al. 2017). Although CEC levels in marine systems are generally lower than those in other aquatic systems (such as rivers or lakes), their presence has been reported in various marine species (field studies). As example, several PCPs have been detected in seafood, for example, BP-3 has been detected at a concentration of up to 98.7 ng·g^−1^ dw, OC up to 103.3 ng·g^−1^ dw, OTNE at 93.5 ng·g^−1^, and galaxolide (HHCB) up to 14,500 ng·g^−1^ (Bachelot et al. 2012; Bayen et al. 2019; Cunha et al. 2018; Pintado-Herrera et al. 2020). BP-3 has been detected at 24.3 ng·g^−1^ and OC at 50.7 ng·g^−1^ (Pico et al. 2019) in fish. OC has been found in a concentration range of 89–782 ng·g^−1^ lipid weight (Gago-Ferrero et al. 2013) and TCS at 0.12–0.27 ng·g^−1^ ww in dolphins (Fair et al. 2009). However, there is still a lack of information regarding bioconcentration for selected PCPs despite their occurrence in the environment. A recent review of UV filters has revealed that, within the timeframe of 2015–2021, only 13 studies assessed the bioaccumulation of BP-3 in wild marine invertebrates (Cuccaro et al. 2022a).

Although PCPs have been well studied in freshwater organisms among different taxa (de García et al. 2017; Kim et al. 2009; Wilson et al. 2003), the information available in the literature related to marine organisms is scarce. Marine bivalves are a target group to assess the bioaccumulation and toxicity of CECs, as they can filter large volumes of water and are susceptible to accumulating chemicals in their tissues. Therefore, these organisms are valuable indicators of aquatic pollution (Aguirre-Martínez et al. 2021; Blasco and Puppo 1999; Dagnino et al. 2007). Bioaccumulation of UV-filters has been recorded in mussels *Mytilus galloprovincialis* after long-term laboratory assays (BP-3 has been found at a concentration of 80 ng·g^−1^ dw, and OC within a range from 327 to 839 ng·g^−1^ dw (Gomez et al. 2012; Vidal-Liñán et al. 2018b). In clams *Ruditapes phillipinarum* exposed to BP-3 and OC, in combination with TiO_2_ nanoparticles, the concentration was 3300 ng·g^−1^ dw and 2000 ng·g^−1^ dw, respectively (Sendra et al. 2017). In addition, in an experiment carried out in fish *Sparus aurata*, the results showed 600 ng g^−1^ for BP-3 (Ziarrusta et al. 2018).

Regarding the antibacterial TCS, a concentration from 0.66 to 881 ng·g^−1^ dw was detected in *M. galloprovincialis* tissues (De Marchi et al. 2020; Freitas et al. 2019; Gatidou et al. 2010; Kookana et al. 2013; Pirone et al. 2019), while in *Perna canaliculus*, TCS has been detected at up to 1000 µg·g^−1^ dw (Webb et al. 2020).

OTNE is among the most-used synthetic fragrances and its presence has been detected in environmental compartments and detected in field studies (Pintado-Herrera et al. 2020). However, there is a gap in the knowledge about its bioaccumulation and its effects in marine organisms (Bester 2009). To our knowledge, only one laboratory study using *R. phillipinarum* as a sentinel organism has tested this compound under a scenario of mixed PCPs (Sendra et al. 2017).

Regarding their toxicity, selected PCPs can provoke alterations in the endocrine system, demonstrating estrogenic activity at certain levels and altering different biological processes (Regnault et al. 2016; Schnitzler et al. 2016; Wang et al. 2018). Studies have revealed BP-3 and OC can alter endocrine or reproduction endpoints in fish (*Oncorhynchus mykiss*, *Oryzias latipes*, and *Danio rerio*), inducing estrogenic effects (Blüthgen et al. 2014; Coronado et al. 2008; Kim and Choi 2014; Kinnberg et al. 2015; Kunz et al. 2006; Rodríguez-Fuentes et al. 2015). Furthermore, they may also induce oxidative stress, activation of biotransformation enzymes, and lipid peroxidation in bivalves (Bordalo et al. 2020; Chaves Lopes et al. 2020; O’Donovan et al. 2020; Sendra et al. 2017; Seoane et al. 2021). Mutagenic and genotoxic responses have also been found under exposure to BP-3 and OC (Cuquerella et al. 2012; Nakajima et al. 2006). Furthermore, TCS has the potential to harm aquatic species including algae, invertebrates, fish, and marine mammals (Bedoux et al. 2012; Dann and Hontela 2011; Tamura et al. 2013). In relation to bivalves, TCS can affect reproductive output and energy-related parameters, induce oxidative stress, lipid peroxidation, and genotoxicity (Binelli et al. 2009; De Marchi et al. 2020; Maynou et al. 2021; Pirone et al. 2019; Rolton et al. 2022; Webb et al. 2020). Studies have shown that exposure to TCS in vivo and in vitro provoked immunosuppression of immune cells of bivalves (Canesi et al. 2007; Matozzo et al. 2012a).

Fragrances, such as HHCB and tonalide (AHTN), have shown to induce oxidative stress and genotoxicity in *R. philippinarum* even at environmental concentrations (Ehiguese et al. 2020); the acute toxicity of these fragrances has been demonstrated in the freshwater mussel *Lampsilis cardium* (Gooding et al. 2006). However, to our knowledge there is not up to date empirical data evaluating the toxicity of OTNE. A theoretical evaluation of the toxicity data concluded that OTNE does not pose an ecological risk to aquatic organisms (McDonough et al. 2017). However, these data are insufficient to conclude that OTNE does not represent any risk to aquatic organisms.

Due to this lack of knowledge, efforts should be made to understand the bioaccumulation and effects of the most frequent PCPs found in environmental compartments in marine sentinel species. In this work, we have hypothesized that bioaccumulation, toxicity, and elimination of PCPs depend on their chemical structure. Therefore, the aims of the present study are i) to evaluate the accumulation and the capacity to reduce PCP concentration over PCP post-exposure period as well as the bioconcentration factor of four selected PCPs [the UV-filters (BP-3 and OC), the antibacterial product (TCS), and a synthetic fragrance OTNE], whose presence has been previously demonstrated in marine systems using *R. philippinarum* as the model organism; ii) to study any toxicological effects of the selected compounds in *R. philippinarum* after a month of exposure and a week of PCP post-exposure period.

## Materials and methods

### Chemicals

Dichloromethane, ethanol, and ethyl acetate were of chromatography quality, purchased from Sigma-Aldrich (Madrid, Spain). Diatomaceous earth (Hydromatrix) was purchased from Agilent Technologies (Madrid, Spain). PTFE centrifuge filters (0.22 μm pore size) were purchased from Ciromfg (FL, USA). Derivatizing agents, N-(tert-butyldimethylsilyl)-N-methyltrifluoroacetamide (MTBSTFA) and acetic anhydride from Sigma Aldrich (Madrid, Spain), were also used to improve the analytical signal. Neutral alumina (58 Å) was used as a sorbent for the clean-up and provided by Sigma-Aldrich (Madrid, Spain). Silica was activated according to Environmental Protection Agency proceedings (3630c, method EPA). Commercial polydimethylsiloxane (PDMS) stir bars [10 mm × 0.5 mm (length × film)] and a 15-position magnetic stirrer were purchased from Gerstel (Germany).

Standards for BP-3, OC, TCS, and the isotopically labeled internal standard benzophenone- 2,3,4,5,6-d5 were purchased from Sigma-Aldrich (Madrid, Spain). OTNE (synthetic fragrance) was purchased from Bordas Chinchurreta Destilations (Seville, Spain). Triclosan-d3 was purchased from LGC Standards (Barcelona, Spain). Table S1 indicates the main physicochemical properties of these PCPs.

### Organisms

Clams, *R. philippinarum*, were obtained from Cetarea del sur, Cadiz (SW Spain). The organisms were acclimated for 7 days in culture tanks (500 L) containing filtered (0.2 μm) natural seawater. The organisms were fed every 2 days with TROPIC MARIN Pro Coral Phyton. The physical–chemical conditions during the acclimation period and experimental period were similar. The water temperature was 17.5 ± 1.2 °C and salinity was between 37 and 38 psu (g L^−1^), dissolved oxygen ranged between 7 and 8.5 mg L^−1^ with a light cycle of 12/12 h light/dark. The filtered seawater of the aquaria was completely changed every 48 h. Minor and major axes of clams were 4.0 ± 0.2 cm and 5.0 ± 0.3 cm, respectively (*n* = 10). A total number of 1200 individuals were transported alive to the laboratory. This species has served as the model organism in several studies due to is wide distribution, easy collection, and sensitivity; moreover, due to its economic value, it is farmed around the world (Aguirre-Martinez et al. 2016; Aguirre-Martinez and Martín-Díaz 2020; FAO 2013; Matozzo et al. 2012b; Moschino et al. 2012).

### Experimental design

The clams employed for experiments were hand collected from the acclimation tank and placed randomly into aquariums before incorporation of PCPs exposure. The experimental design consisted of six groups; with four replicates in the four PCPs tested and two replicates for control and solvent control. The concentration employed was 10 µg L^−1^ for TCS, OTNE, BP-3, and OC (nominal concentration). The solvent concentration was 0.004% ethanol in all tanks, except in the water controls. The nominal concentrations were chosen to address environmental levels and effect concentrations (Pintado-Herrera et al. 2020; Tsui et al. 2019). After the acclimation period (7 days), the organisms (*n* = 50) were exposed to TCS, OTNE, BP-3, and OC for 26 days. Five sampling periods were selected to measure endpoints: 1 day before starting the experiment, day 2, day 7, day 14, and day 26. Furthermore, a PCP post-exposure period of 7 days was also included.

Seven individuals per replicate tank were collected at each sampling time and dissected. The experimental conditions are detailed in Table S2. The shells were discarded and the soft bodies were used for bioaccumulation analysis (4) and for biomarker analysis (3). The digestive glands were dissected and frozen in liquid nitrogen and stored at – 80 °C until biomarker analysis. Aqueous samples were also collected at six different time intervals (2 h, 5 h, 24 h, and 48 h) between consecutive water changes to measure any changes in the concentrations of the target compounds.

### Clam tissue extraction

Extraction of the analytes from clam samples was achieved by pressurized liquid extraction (in-cell PLE), using an accelerated solvent extractor ASE 200 unit from Dionex (Sunnyvale, CA, USA), with 11-mL stainless-steel cells. A pool of each tank (4 clams per tank) was considered for a tissue sample. Briefly, a cellulose filter was placed on the bottom of each cell, 2 g of activated silica, to separate the analytes from interfering compounds, dried and milled solid samples (1 g) were homogenized with 0.5 g of silica to fill the extraction cell; and then a cellulose filter was placed between the different layers in the cell. Under optimal conditions, dichloromethane was used as the solvent, in three static extraction cycles of 5 min, at 100 °C and 1500 psi with a purge time of 60 s and a flush volume of 60%. The purification of the extracts was performed simultaneously to the extraction (in-cell clean-up) by adding the sorbent into the cell (activated silica). Finally, the extracts (30 mL) were evaporated to dryness using a Syncore Polyvap (Büchi, Switzerland) and re-dissolved in 500 µL of ethyl acetate. They were then centrifuged at 10,000 rpm to remove possible interferences, and finally the extracts were filtered with a PTFE filter and derivatized with MTBSTFA; 10 µL left for 30 min at room temperature (conditions previously optimized by the group). This process was performed using a modification of Pintado-Herrera et al. (2016).

### Water sample extraction

The water samples were collected after 2, 5, 24, and 48 h of exposure prior to renewal of water (sampling period: days 0–2). Twenty-milliliter amber-glass bottles were used for the sampling; all spiked tanks were sampled (16 tanks). All glassware material was cleaned with deionized water and ethanol and baked at 500 °C (for 4 h) prior to use to avoid any possible contamination of the samples. Duplicates of 10 mL were used for the analysis. All the samples were analyzed within 24 h after collection, to avoid possible degradation or adsorption into the walls of the bottle. Stir bar sorptive extraction technique (SBSE) was used for the extraction of the samples, according to Pintado-Herrera et al. (2014). Stock standard solutions of each compound were prepared in ethanol.

### PCP analysis

The separation, identification, and quantification were performed using gas chromatography (SCION 456-GC, Bruker) coupled to a triple quadrupole mass spectrometer, specifically a SCION (Bruker), with a CP 8400 Autosampler. Capillary gas chromatography analysis was carried out in a BR-5 ms column (30 m × 0.25 mm i.d. × 0.25 μm film thickness), keeping the helium carrier gas flow at 1 mL min^−1^ and the transfer line and the injection port temperature at 280 °C. The column temperature ramp was as follows: 70 °C for 3.5 min, increased at 25 °C min^−1^ to 180 °C, then at 10 °C min^−1^ to 300 °C, and held for 4 min. The injection volume was 1 μL with splitless mode for GC and the solvent delay was 4.5 min. Electron ionization (EI) mode was set at 70 eV. The collision gas was argon with a pressure of 2 mTorr.

The mass detector was operated with multiple reaction-monitoring (MRM) mode. The identification and quantification of target compounds were based on comparing retention times and two transitions of each analyte (one for quantification and one for confirmation) to those for commercially available pure standards. Calibration curves were constructed to measure levels of the selected analytes in the clam samples in the range of 5–500 ng g^−1^ and in the water samples between 0.01 and 10 µg L^−1^. Internal standards (BP-2,3,4,5,6-d_5_ and TCS-d_3_ at 50 ng g^−1^) were added to the vials before injection to correct possible fluctuations in the MS signal (ion suppression), comparing the signal intensity of the internal standards spiked in the calibration curve and in the sample extracts. All data were processed using the Bruker MS Workstation software. Further details on GC–MS/MS analysis can be found in Pintado-Herrera et al. (2016).

### Bioconcentration factor (BCF)

The bioconcentration factor (BCF) assesses the degree to which the organism accumulates a selected compound from the environment only through its respiratory and dermal surfaces (Arnot and Gobas 2006), and it can be estimated under controlled laboratory conditions. This factor depends, among other factors, on the type of organism or the duration of the exposure time. Several methods to estimate BCF have been published, for example, from the octanol/water partition coefficient (*K*_ow_) (Meylan et al. 1999); also, the static or the dynamic BCF could be calculated, the latter methodology is considered more realistic as it takes into account the post-exposure rate (Eq. 1).

1 $${\text{d}}{C}_{{\text{clams}}}/{\text{d}}t={k}_{uptake}\times {C}_{{\text{w}}}\left(t\right)-{k}_{post-exposure}\times {C}_{{\text{clams}}}\left({\text{t}}\right)$$

where *C*_clams_ is the concentration in the organism, *k*_*uptake*_ is the uptake rate constant from the water, *C*_w_ is the concentration in water, *t* is the time, and* k*_post-exposure_ is the post-exposure rate constant.

Furthermore, experimental BCF results were compared to BCF values estimated by using quantitative structure–activity relationships (QSAR) models. These models have been previously applied in several studies where log *K*_ow_ is used to predict the BCF of organic contaminants (Arnot and Gobas 2006; Donkin et al. 1991; Mackay 1982) and the relation proposed by the Technical Guidance Documents (TGD) on risk assessment (European Commission 2003) (see equations in Table S3).

### Biochemical responses

The digestive glands of three clams from each tank were collected, homogenized, and extracted following the methodology described by Lafontaine (2000) (ratio of 1:5 wet weight/buffer volume). The homogenization processes were carried out using an Ultra Turrax (VWR Vos 14). The homogenized tissues were centrifuged at 15,000 g for 30 min at 4 °C and stored at − 80 °C. The supernatant fractions (S15) were collected for the quantification of total proteins (TP), ethoxyresorufin O-demethylase (EROD), glutathione S transference (GST), antioxidant enzyme activities, lipid peroxidation (LPO), and DNA damage. The biochemical analyses were determined using a microplate reader (TECAN Genios). The TP content was measured following an adaptation of Bradford’s methodology using bovine serum albumin (BSA) as the standard (Bradford 1976). All analyses were performed in triplicate.

The EROD activity assay was adapted from fingerling rainbow trout (Gagné and Blaise 1993) to clams. Fifty microliters of the S15 fraction was added to 160 μL 7-ethoxyresorufin in dark microplates (96 flat-bottom well). Then 10 μL of reduced NADPH was added to each well to initiate reaction, and 7-ethoxyresorufin was detected fluorometrically every 10 min for 60 min at 30 °C, 516 nm (excitation) and 600 nm (emission) filters. Calibration was achieved using a standard calibration curve of 7-hydroxyresorufin. The results were standardized to total protein (TP) content and expressed as pmol·min^−1^·TP.

GST activity (EC 2.5.1.18) was calculated following McFarland et al. (1999) using 1-chloro-2, 4-dinitrobenzene (CDNB) as a substrate. Absorbance was recorded at 340 nm. The results were expressed as mmol CDNB conjugate formed min^−1^ mg^−1^ TP.

Superoxide oxidase activity (SOD, EC 1.15.1.1) was determined using an SOD assay kit-WST following the manufacturer’s instructions (Dojindo Molecular Technologies, Inc., Kumamoto, Japan). SOD activity was measured by mixing the reagents from 220 μL of the WST kit with 20 μL of the sample. After incubation for 20 min at 37 °C, the absorbance was measured at 450 nm. The results were expressed as U SOD mg^−1^ TP. One international unit is equal to 1/60 μkat, and one katal is the amount of enzyme that converts 1 mol of substrate per second.

Catalase activity (CAT, EC 1.11.1.6) was measured using the decrease in absorbance due to the consumption of H2O2 at 240 nm according to Beers and Sizer (1952) and validated for microplate assay (Li and Schellhorn 2007). Results were expressed in U CAT mL^−1^ mg^−1^ TP.

Total glutathione peroxidase (T-GPx, EC 1.11.1.9) and Se-dependent glutathione peroxidase (Se-GPx, EC.1.11.1.9) were measured following the methodology described by Flohé and Günzler (1984) and adapted by McFarland et al. (1999). Absorbance was measured at 340 nm and determined by means of the increase in the NADPH oxidation, while using cumene hydroperoxide and hydrogen peroxide as the substrates, respectively, Activities for both enzymes were expressed as nmol NADPH min^−1^ mg^−1^ TP.

Glutathione reductase (GR, EC 1.8.1.7) was determined following the methodology of McFarland et al. (1999) modified from Cohen and Duvel (1988). Briefly, the assay mixture (AM) consisted of 200 mM sodium phosphate buffer (pH 7.6), containing 1 mM oxidized glutathione (GSSG) and 0.1 mM NADPH. Then 20 μL blank (homogenization buffer) or the sample was added to the wells. The reaction was initiated by the addition of 200 μL of the 25 °C preheated AM, then loss of NADPH was recorded every 60 s during 10 min at 25 °C. Absorbance was measured at 340 nm. The results were expressed as nmol NADPH min^−1^ mg^−1^ TP.

Lipid peroxidation (LPO) was measured employing an adaptation of the thiobarbituric acid reactive substance (TBARS) method of Snell (1987). Oxidative stress leads to the production of malondialdehyde (MDA) resulting from the degradation of the initial products of free radical attacks on fatty acids (Janero 1990). MDA reacts with 2-thiobarbituric acid producing tetramethoxypropane (TMP) which can be measured spectrophotometrically. Standards and samples were incubated in Unitronic 320 OR P Selecta Heaters at 70 °C for 10 min. Absorbance was measured at 540 nm, in order to set the standard curve of TMP enabling the indirect determination of MDA. LPO was expressed as μg TBARS mg^−1^ TP.

DNA damage was estimated using the DNA precipitation assay described by Olive (1988); this methodology is based on the K-SDS precipitation of DNA–protein crosslink, which uses fluorescence to quantify the DNA strands (Olive et al. 1988; Gagné et al. 1995). A volume of 25 μL of the homogenate was mixed in Eppendorf vials with 200 μL of SDS 2% (10 mM EDTA, 10 mM Tris base, and 40 mM NaOH) for 1 min. Then, 200 μL of KCl (0.12 M) was added, and the solution heated at 60 °C for 10 min, mixed, and cooled at 4 °C for 30 min in order to precipitate the genomic DNA linked to SDS-associated nucleoproteins. The mixture was then centrifuged at 8000 g for 5 min (4 °C). Fifty microliers of the supernatant was added to 150 μL of Hoescht dye 0.1 mg mL^−1^ (diluted in buffer containing 0.4 M NaCl, 4 mM sodium cholate, and 0.1 M Tris–acetate, pH 8.5) in a dark microplate (96 flat-bottom wells). Fluorescence was measured using 360 nm (excitation) and 450 nm (emission). Salmon sperm genomic DNA standards procured from Sigma Aldrich were used for DNA calibration. Results were expressed as μg DNA mg^−1^ TP.

### Data analysis

Kinetic curves were fitted using the SciDAVIs 2.3.0 free software and applying the Levenberg–Marquardt algorithm with a tolerance of 0.0001. Graphical plots and statistical analyses of biomarkers were carried out using the SigmaPlot 11.0 software. One-way ANOVA with a Dunnett post hoc at *p* < 0.05 was performed to assess all the responses.

## Results and discussion

### Clam tissue extraction validation

The recovery percentages were higher than 80% for the organic compounds analyzed (Table S4). In the blank samples, performed in the same way as the samples but without the matrix, values lower than the method limit of detection (MDL) were detected except for OC for which up to 8.5 ng g^−1^ was measured. Method limits of detection (MDLs) ranged between 0.07 and 0.1 ng g^−1^ for the target compounds, defined for a signal-to-noise ratio of 3. The suppression due to matrix interferences, considered for each sample by means of internal standards, was between 10 and 38%. Finally, the reproducibility of the method, calculated in spiked clams on different days, was 93% (*n* = 3).

### Accumulation of PCPs tested over exposure and post-exposure period

Although there is now strict legislation concerning UV-filters and musks in the European Union (1223/2009/UE), and recently new concentration limits have been published for BP-3 and OC (2022/1176/UE), in relation to manufacture and utilization (Sánchez-Quiles and Tovar-Sánchez 2015), no environmental legislation has been established for the most frequent PCPs found in natural environment. In this regard, some UV filters such as 2-ethylhexyl 4-methoxycinnamate (EHMC), OC, and BP-3 have been recently included on European Watch Lists (2015/495/EU and 2022/1307/EU) as pollutants to be monitored in surface water. For the antibacterial TCS, its use has been already regulated by the European Commission from the beginning of 2017 for selective products (e.g., maximum of 0.2% in mouthwashes) (2016/110/EU).

Although the four compounds selected in this study have been reported in aquatic systems reaching levels of a few parts per billion in some places (Cadena-Aizaga et al. 2022), the seawater employed in these experiments and obtained from a well did not show background concentrations, except for OC (0.25 µg·L^−1^ for OC).

The water concentration of target analytes was measured in treatment tanks with the organisms before water renewal. Two hours after spiking, the concentrations of the analytes were 9.5 ± 1.4 µg L^−1^ for BP3, 7.7 ± 1.5 µg L^−1^ for OC, 5.4 ± 1.1 µg L^−1^ for TCS, and 3.8 ± 0.8 µg L^−1^ for OTNE. After 48 h, the chemical compounds showed a loss of 66, 96, 94, and 95% for BP-3, OC, TCS, and OTNE, respectively (Table S5). There may be different reasons for this, including degradation and volatilization processes of these compounds (Ozaki et al. 2021), adsorption into the walls of the aquariums, bioaccumulation of hydrophobic organic chemicals (Lietti et al. 2007; Vidal-Liñán et al. 2018), or bacterial degradation (Dhillon et al. 2015).

Accumulation data of PCPs in the organisms from the control and the solvent control tanks were measured in the same range to those measured at the beginning of the experiment (day 0); it was lower than 43.4 ng g^−1^ dw for the four chemicals (Table S6).

**Fig. 1:**
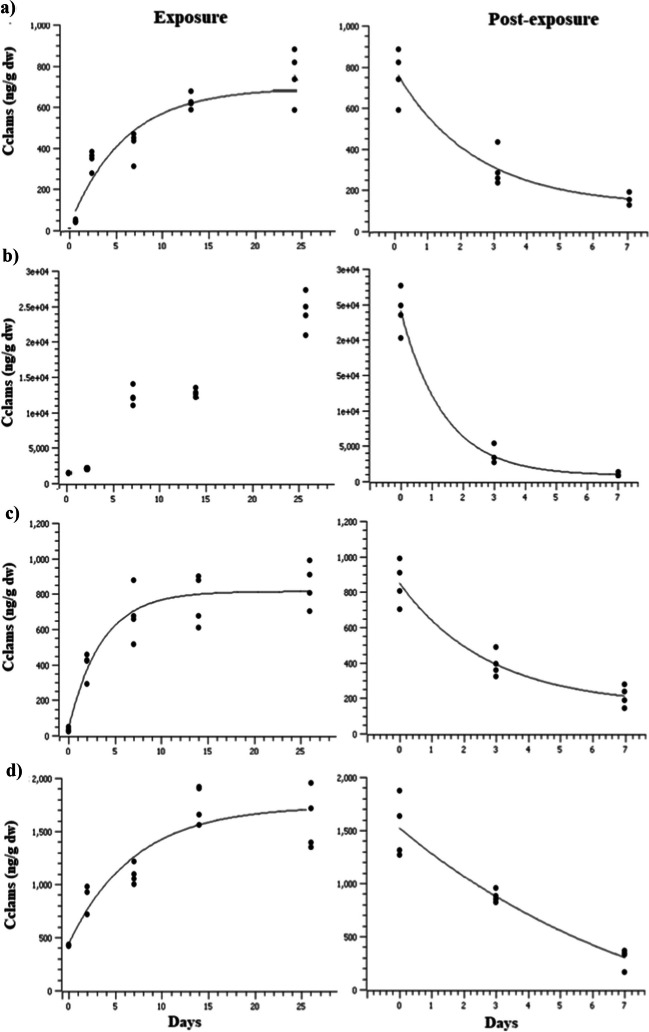
Profile of accumulation and post-exposure phases in *R. philippinarum* exposed to **a** OTNE, **b** BP-3, **c** OC, and **d** TCS. Lines correspond to the expected values based on the model calculations and (●) correspond to the experimentally determined values (*n* = 4)

The experimental and model kinetic data of the selected compounds are shown in Fig. 1. The results of PCPs’ accumulation over exposure and post-exposure period by clams are provided in Table S7. OTNE, TCS, and OC reached a steady-state condition following first-order kinetics (*r*^2^ > 0.82 for the uptake period and *r*^2^ > 0.91 for the post-exposure period). BP3, which is the less apolar compound, did not reach a steady state at the end of the uptake period and the experimental data were not consistent with this model.

Significant differences (*p* < 0.05) were found between solvent control and all uptake periods (days 2, 7, 14, and 26 of exposure). Furthermore, a fast increase was observed at the beginning of the experiment; however, after day 7 the rate of accumulation decreased to a slower pace until stabilization. This trend has been observed in other organic chemicals such as 4-n-nonylphenol, bisphenol A, and tris-(2-chloroethyl) phosphate (Garcia-Galan et al. 2017; Gatidou et al. 2010; Ismail et al. 2014). At the beginning of the experiment (day 0), the levels of PCPs in the exposed clams were lower than 36 ng·g^−1^ dw, but after 2 days of exposure, the levels of the selected chemicals in the tissues increased for all compounds: OTNE (334 ng g^−1^ dw), OC (398 ng g^−1^ dw), TCS (540 ng g^−1^ dw), and BP-3 (733 ng g^−1^ dw). After 26 days of exposure, the concentration in the clams reached up to values of 682 ng g^−1^ dw (OTNE) < 806 ng g^−1^ dw (OC) < 1523 ng g^−1^ dw (TCS) < 24,058 ng g^−1^ dw (BP-3). The concentration values of chemicals measured during each sample period are indicated in Table S7.

In comparison to experimental data from other studies, Vidal-Liñán et al. (2018) reported data for OC (833 ng·g^−1^ dw) and BP-3 (59 ng·g^−1^ dw) in *M. galloprovincialis* after 30 days of exposure at 1 µg L^−1^); and for TCS 881 ng·g^−1^ dw was measured in *M. galloprovincialis* after 28 days of exposure at 305 ng L^−1^ (Gatidou et al. 2010); 13.88 ng·g^−1^ after 28 days exposed at 1 µg L^−1^ (Pirone et al. 2019); 1000 µg g^−1^ dw in *P. canaliculus* after 48 h exposed at 0.2 mg L^−1^ (Webb et al. 2020). Some differences were observed regarding the exposure periods, especially for BP-3, in the concentrations registered in the present study in *R. philippinarum* for similar periods.

The present study found that OTNE bioconcentration in clams reached similar values (520 ng·g^−1^ dw) after 14 days of exposure compared with those reported by Sendra et al. (2017) for the same species, following a similar methodology but a different mixture of PCPs and nanoparticles.

Regarding the accumulation and following a first-order kinetic model, the results for the uptake rate ranged between 196.8 and 467.3 L kg^−1^ day^−1^ (Table S8); these values are higher than those reported for *M. galloprovincialis* 93 L kg^−1^ day^−1^ for TCS (Gatidou et al. 2010) and 281.7 L kg^−1^ day^−1^ for OC (Vidal-Liñán et al. 2018).

It was observed that the levels of PCPs accumulated in the tissues decreased during the post-exposure period, although they did not return to the initial levels (Table S7). At the end of the post-exposure period, the percentages were 68% for OC, 79% for OTNE, 85% for TCS, and 94% for BP-3. The levels in the clam tissues decreased after the post-exposure period in relation to accumulation after 26 days of exposure to PCPs: OC < OTNE < TCS < BP-3 with values of a 4.03-, 4.87-, 5.07-, and 24.77-fold changes, respectively. According to the kinetic model selected, the results for *k*_post-exposure_ ranged from 0.212 to 0.416 day^−1^ (Table S8), BP-3 was the compound that presented the highest elimination rate (0.416 day^−−^). Compared with previously published data, data for TCS and OC were only found by estimating a *k*_depur_ of 0.056 day^−1^ for TCS and 0.13 day^−1^ for OC in mussels, even at longer post-exposure period times than in our study (up to 28 days) (Gatidou et al. 2010; Vidal-Liñan et al. 2018). The half-life of PCPs over post-exposure time (*t*_1/2_ = ln2/*k*_post-exposure_) ranged between 1.6 and 3.2 days in the same order as *k*_depur_. These differences in the parameters estimated may be for different reasons such as the organisms themselves, the experimental conditions, and extraction techniques, among others.

Furthermore, considering the results obtained from the biconcentration experiment, it should be emphasized that even with an effective post-exposure rate (up to 94% for BP-3), some alterations could be taking place in the organisms. A battery of biomarkers was measured to evaluate these alterations. The results are shown in the section “Biomarker response.” Additionally, some biotransformation processes may generate metabolites, as has already been demonstrated in other biological matrices for BP-3, OC, and TCS (Chiriac et al. 2022; Saunders et al. 2020; Weatherly and Gosse 2017).

### BCF estimations

Being the dynamic BCF the ratio between *k*_uptake_ and *k*_post-exposure_, the dynamic BCF values ranged from 2204 to 852 L kg^−1^ (log BCF from 3.34 to 2.93) (Table S8). The highest BCF value was obtained for OC, the most apolar compound (log *K*_ow_ 6.8).

The log BCF estimated for TCS in this study is in the same order of magnitude as the data previously reported in other aquatic species, for example, Escarrone et al. (2016) reported a value of 2.5 in *Poecilia vivipara* and Kookana et al. (2013) estimated a value of 2.81 in *M. galloprovincialis*. Vidal-Liñán et al. (2018) reported BCF values in mussels of 2210 L kg^−1^ for OC (log BCF 3.34), in agreement with our study, although in the same study, no increase in the accumulation of BP3 was observed during exposure time. Regarding UV filters, BCF has also been estimated in other aquatic organisms, for example, in *O. mykiss* 1267 L kg^−1^ BCF (log BCF 3.10) (Saunders et al. 2020), in *D. rerio* BCF values up to 858 L kg^−1^ for OC (Pawlowski et al. 2019), and 94 L kg^−1^ for BP3; although in this last study, non-dynamic BCF were calculated since there was no post-exposure period (Blüthgen et al. 2012).

For OTNE, the BCF calculated in this study was 1589 L kg^−1^ (log BCF 3.20); however, it is difficult to compare this data since, to our knowledge, no previous data for experimental BCF have been published. Only data from field studies report the importance of increasing the knowledge about this fragrance (Biel-Maeso et al. 2019; Klaschka et al. 2013; Pintado-Herrera et al. 2020). However, log BCF values for other polycyclic musks with similar physicochemical properties (e.g., HHCB and AHTN) were calculated for two benthic organisms (*Chironomus riparius* larvae and *Lumbriculus variegatus*) which determined experimental values of 1.7 and 3.84 for AHTN, and 1.93 and 3.59 for HHCB, for the larvae and the worm, respectively. Although the values between these organisms may be different, and there was no post-exposure period in this study, these values are in concordance with the values calculated in our study, especially for the worm. In addition, our BCF data were in the same range as bioaccumulation data (BAF, bioaccumulation factor) detected for HHCB in zebra mussels from the USA (BAF = 2610–4890, log BAF 3.41–3.68) (Reiner and Kannan 2011).

Regarding our results and according to the REACH legislation, chemicals with BCF values higher than 2000 L·kg^−1^ are considered bioaccumulative. In this sense, only the UV filter OC may be considered bioaccumulative (ECHA 2023). Furthermore, OC bioaccumulation has been demonstrated in aquatic organisms from different trophic levels (Pawlowski et al. 2019; Gago-Ferrero et al. 2013).

Finally, log BCF values were also estimated using various QSAR linear models, applying equations from the literature (Table S3). For compounds with a log *K*_ow_ higher than 6 (i.e., OC), the bilinear equation is expected to provide more accurate data due to the cut-off problem identified for hydrophobic substances, as recommended by the European Commission on risk assessment (European Technical Guidance 2003). Nevertheless, when comparing our experimental BCF data to values calculated using the aforementioned QSAR equations, significant differences between calculated and experimental data were observed, particularly for OC—the most hydrophobic compound—despite the use of bilinear equations (Table S8). To conclude, for this compound models only based on the *K*_ow_ parameter overestimated the log BCF values, highlighting the limitations of these equations. Nonetheless, these equations are a valuable tool when no experimental data are available, as they consider only *K*_ow_ and no other processes, such as metabolism or other types of interactions, are required.

### Biomarker responses

**Fig. 2:**
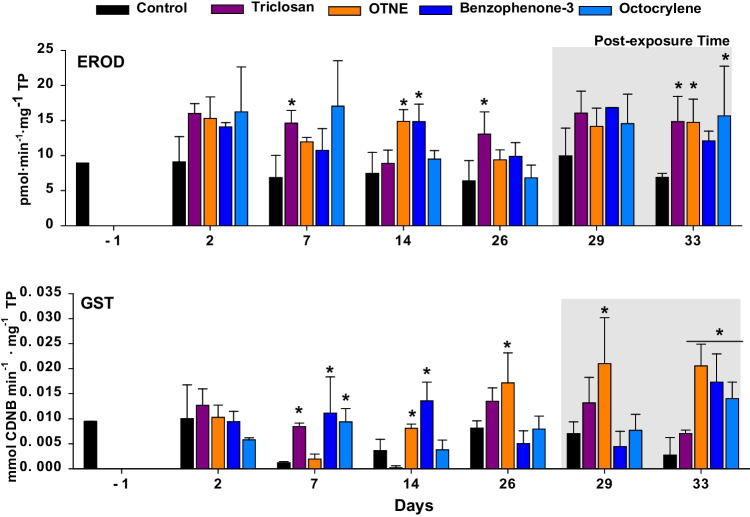
Biomarkers of phase I, ethoxyresorufin O-deethylase (EROD), and phase II, glutathione S-transferase (GST), measured in digestive gland tissues of *R*. *philippinarum* exposed 26 days to experimental treatments including control (seawater) and PCPs and 7 subsequent days of post-exposure. Data are given as mean ± standard deviation. Asterisks indicate significant differences from control (one way ANOVA, *p* < 0.05)

**Fig. 3:**
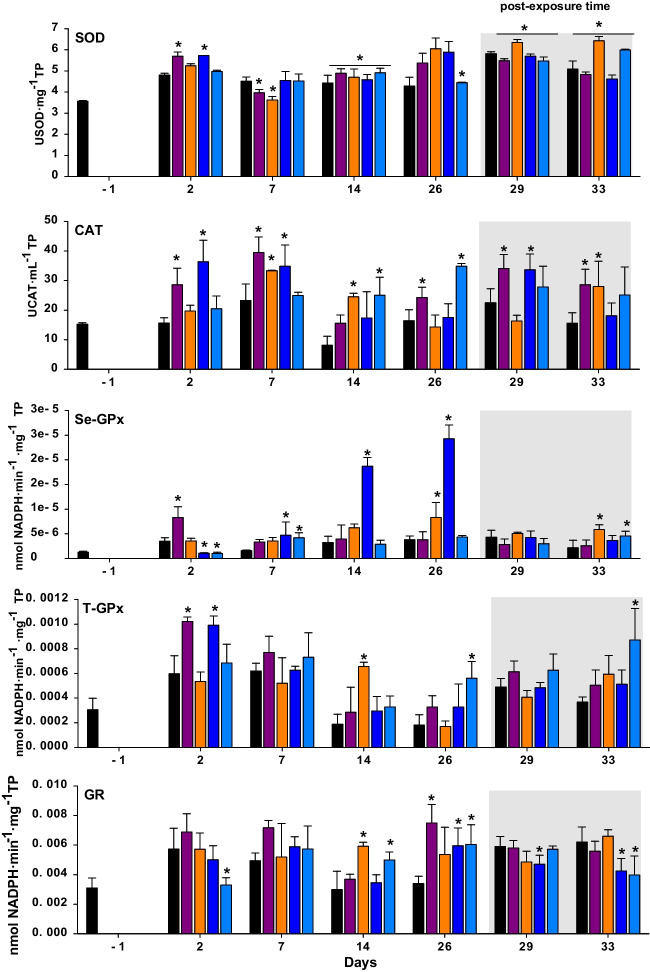
Antioxidant biomarkers of exposure and effect including superoxide dismutase (SOD), catalase (CAT), Se-GPX total glutathione peroxidaxe (T-GPx), and glutathione reductase (GR) measured in digestive gland tissues of *R. philippinarum* exposed 26 days to experimental treatments including control (seawater) and PCPs and 7 subsequent days of post-exposure. Data are given as mean ± standard deviation. Asterisks indicate significant differences from control (one way ANOVA, *p* < 0.05)

Mortality was not significant over the exposure and post-exposure periods; it was lower than 5% in all treatments. Similarly, selected compounds in adult bivalves from other studies did not show significant mortality (Falfushynska et al. 2021; Santonocito et al. 2020). Nevertheless, in a previous work with *M. galloprovincialis* larvae, an EC50 of 3472.59 µg·L^−1^ was established for BP-3 (Paredes et al. 2014) and 213 µg·L^−1^ for TCS (Cortez et al. 2012; Rolton et al. 2022; Tato et al. 2018), demonstrating that the organism’s development stage is key to assess the mortality of bivalves regarding PCP compounds. In addition, these compounds could also affect other species at the early developmental stage. An EC50 of between 567 and 1091 μg·L^−1^ was reported for the sea urchin *Paracentrotus lividus* exposed to OC (Giraldo et al. 2017). Although no lethal effect was observed in *R. philippinarum* in this study, significant changes in enzyme activities related to the mechanisms of xenobiotic metabolisms were observed (Figs. 2 and 3).

Regarding phase I, the clams exposed to TCS, BP-3, and OTNE showed significant differences in EROD activity from day 7 to 26 (*p* < 0.05), and even after a week of post-exposure (*p* < 0.05); TCS was the first PCP to activate the detoxification process among the compounds tested. Digestive gland tissue is the main site for increased EROD activity. EROD induction revealed the presence and availability of PCPs during the experiment (Aguirre-Martínez and Martín Diaz 2020); moreover, it has been demonstrated in other laboratory studies that PCPs induce EROD activity in this species (Aguirre-Martínez 2016). Similarly, the study by Sendra et al. (2017) found that EROD activity increased under the same conditions when clams were exposed to TiO_2_ (nano and bulk) with these four PCPs.

In the work of Falfushynska et al. (2021), *M. edulis* were exposed to 10 and 100 µg·L^−1^ of UV-filters (OC) for 14 days. The lowest concentration did not show significant changes in EROD activity in the digestive gland; however, a significant decrease was observed at 100 µg·L^−1^. Considering these results, a high concentration of UV-filters (such as OC) can provoke a suppression of the phase I enzyme activity; thus, activation was observed later during the post-exposure time. This suppression of enzyme activity was also observed during the metabolization of organic compounds in *M. galloprovincialis* exposed to higher concentrations of BP-3 (1 µg·L^−1^) (Bordalo et al. 2020).

GST is a biomarker of phase II that allows the biotransformation and disposal of exogenous compounds such as PCPs (Contreras-Vergara et al. 2004). The GST enzyme acts as a defense against oxidative stress and it is essential for the elimination of intercellular ROS in marine organisms (De Lafontaine et al. 2000). The GST enzyme was activated after 7 days of exposure, where a significant increase was observed in the clams exposed to TCS, BP-3, and OC (Fig. 3; *p* < 0.05). At the end of the exposure time, OTNE was the only compound, which was able to increase GST activity. During the post-exposure period, OTNE showed a significant increase after 3 days (Fig. 3; *p* < 0.05), and after a week of post-exposure, a significant increase in GST activity was found for clams exposed to OTNE, BP-3, and OC (*p* < 0.05). The exposure to OTNE, BP-3, and OC may have caused the xenobiotic biotransformation system of the bivalves to become dysregulated due to the opposing effects of these compounds on phase I and phase II biotransformation enzymes as was investigated in the work of Falfushynska et al. (2021). Increases in the GST enzyme and gene expression in different bivalve tissues and immune cells exposed to UV-filters (Santonocito et al. 2020) and TCS (Canesi et al. 2007; De Marchi et al. 2020; Parolini et al. 2013) have been demonstrated in previous works. In the work of Santonocito et al. (2020), a significant reduction in gene expression after the no-exposure period was observed; therefore, 3 days of post-exposure was enough to restore the homeostasis of the organism. In the present work it was observed that even after a week of post-exposure, the homeostasis of the clams was not restored under OTNE, BP-3, and OC. Therefore, the exposure time could determine irreversible changes or the need for extra time to restore xenobiotic metabolism.

GPx and GR are antioxidant enzymes that join in the conjugation of xenobiotics with an endogen compound and the reduction of xenobiotics to produce oxygen free radicals (Hartman et al. 2021). The activity of glutathione reductase (GR, an enzyme involved in glutathione recycling) was suppressed in the clams exposed to OC and BP-3 after 2 days of exposure (in this period only OC showed significant differences from control) and a week of post-exposure. Furthermore, after 26 days all the compounds showed a significant increase in GR activity with the exception of OTNE (Fig. 3; *p* < 0.05). Similarly, an increase in GST and suppression of GR activity has been reported in fish exposed to UV filters (Campos et al. 2017; Grabicova et al. 2013). The suppression of GR activity can limit the amount of glutathione-GSH that serves as an essential co-substrate for GST and could therefore counteract the increase in GST activity observed (Gupta et al. 2016).

On the other hand, Se-GPx activity showed significant changes under PCP exposure (Fig. 2). After 2 days of exposure, TCS showed a significant increase in Se-GPx in relation to the controls (*p* < 0.05), while a significant decrease was found in the clams exposed to both organic UV-filters (*p* < 0.05). UV filters showed a significant increase from day 7 until the end of the exposure time (*p* < 0.05). In general, the literature reveals the activation of antioxidant enzymes in bivalves’ digestive glands when exposed to BP-3 (Falfushynska et al. 2021; Seoane et al. 2021), the immune cells (Canesi et al. 2007), the digestive glands (Binelli et al. 2011; Matozzo et al. 2012a; Riva et al. 2012), and soft tissues (Parolini et al. 2013) of mussels exposed to TCS.

After a week of post-exposure, the clams exposed to OTNE and OC showed an increase in Se-GPx (*p* < 0.05; Fig. 2). In the study of Santonocito et al. (2020), it was demonstrated that *R. philippinarum* exposed to UV filters (4-MBC) induced the gene expression of antioxidant enzymes (GPx and CAT) after 7 days of exposure and 3 days of post-exposure. The regulation of antioxidative enzymes over post-exposure may indicate that a post-exposure of 1 week is not enough time for these compounds to compensate for the oxidative stress experienced. The antioxidant CAT activity evaluated in the present study showed a significant increase with respect to the controls for all the compounds tested from day 2 of exposure to day 26 (*p* < 0.05; Fig. 2). TCS and BP-3 were the first and the most potent PCPs to increase the CAT activity after 2 days of exposure until the end of exposure time (*p* < 0.05); these two PCPs (TCS and BP-3) were also modulated during the post-exposure time (*p* < 0.05). The antioxidant SOD showed significant differences among treatments and controls over the exposure and post-exposure times (Fig. 2). Significant increases and decreases of SOD (*p* < 0.05) were found from day 2 after TCS and BP-3 exposure, and all the compounds showed modulation after 14 days. Increases and decreases in SOD were observed according to the sampling time and the metabolism of each PCP tested. An increase in SOD activity was found in clams exposed to TCS during both post-exposure times (*p* < 0.05), while a significant decrease was found for the rest of the PCPs (*p* < 0.05).

**Fig. 4:**
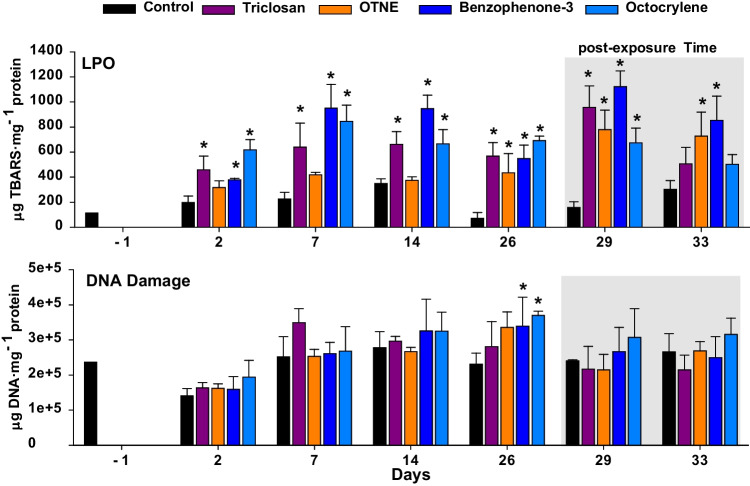
Changes in lipid peroxidation (LPO) and DNA damage measured in digestive gland tissues of *R. philippinarum* exposed 26 days to experimental treatments including control (seawater) and PCPs and 7 subsequent days of post-exposure. Data are given as mean ± standard deviation. Asterisks indicate significant differences from control (one way ANOVA, *p* < 0.05)

Some changes in the levels of antioxidant enzymes can provoke unwanted effects such as LPO and DNA damage (Fig. 4). LPO showed a significant increase from the second day of exposure to TCS and both UV-filters (BP-3 and OC) until the last day of the exposure time (Fig. 4; *p* < 0.05). However, OTNE only showed a significant increase at 26 days of exposure (*p* < 0.05). LPO is a reliable response to assess oxidative stress induced by xenobiotics. For instance, in the work of Pirone et al. (2019), changes in the regulation of antioxidant enzymes in *Mytilus galloprovincialis* exposed to very low concentration of TCS were not observed; however, a significant increase in LPO was revealed. There was an increase in the LPO levels observed in bivalves exposed to UV-filters (Seoane et al. 2021) and TCS (De Marchi et al. 2020; Webb et al. 2020) during long-term experiments. LPO is a robust measure, but oxidative stress is also sometimes evidenced in certain tissues. For example, the LPO measured in *Amarilladesma mactroides* exposed to 1 μg·L^−1^ BP-3 after 96 h showed significant differences with respect to the controls only in the mantle tissues (Chaves Lopes et al. 2020).

A previous work exposed *Patinopecten yessoensis* to BP-3 for 45 days, the results did not show any differences for CAT, SOD activities, ROS, and even LPO; however, the authors found a relevant endpoint to confirm biological damage such as the response increase in Na + /K + -ATPase activity accompanied by a decrease in ATP content (Liao et al. 2019).

In the present study, after 3 days of post-exposure, all the chemical compounds showed a significant increase in LPO levels (Fig. 4; *p* < 0.05). However, significant differences were found for OTNE and BP-3 (*p* < 0.05) after a week of post-exposure. Therefore, the biological memory may show the consequences of previous PCP exposure time even after the exposure time. Lipid metabolism has been recently identified as an important target for toxicity in lipophilic UV filters (Blüthgen et al. 2014; Falfushynska et al. 2021; Stien et al. 2020, 2019).

Our data indicate a significant increase in DNA damage after 26 days of exposure induced by both UV-filters (Fig. 4; *p* < 0.05); similar results have been recently observed in bivalves exposed to different UV filters by other studies (Cuccaro et al. 2022b; Falfushynska et al. 2021; Santonocito et al. 2020). Although our results did not show DNA damage after TCS and OTNE exposure, previous works have demonstrated genotoxicity in *R. philippinarum* (Matozzo et al. 2012a) and *Dreissena polymorpha* hemocytes (Binelli et al. 2009; Parolini et al. 2013) exposed to environmentally relevant concentration of TCS.

Not much data have been published regarding OTNE toxicity; some experiments have observed mortality, adverse effects on reproductive capacity, and morphological changes in fish and the crustacean *Daphnia magna* (McDonough et al. 2017). However, many biological processes were not studied under the effects of OTNE. Even so, some works with other fragrances such as HHCB and AHTN have demonstrated changes in phase I, phase II, antioxidant enzymes, LPO and DNA damage, immunotoxicity, and neurotoxicity at environmentally relevant concentrations (Ehiguese et al. 2020).

Our data have revealed that the toxicity of selected PCPs at environmental concentrations was not directly related to lipophilicity or bioconcentration. This study underlines the importance of performing bioassays to assess the toxicity of emerging contaminants in aquatic biota with special interest in sentinel organisms such as bivalves.

## Conclusions

The bioaccumulation of the four PCPs selected under laboratory conditions has been reported. OTNE and OC presented higher uptake rates. The highest removal rate was found for BP-3, although the final concentration levels were higher than the concentrations from non-exposed organisms. It was concluded that those chemicals with higher log *K*_ow_ matched the higher BCF in this study. Moreover, it was observed how the experimental conditions and the organism selected might influence the results, making comparison between studies difficult. Additionally, results from this study provide new information concerning TCS, BP-3, and OC for the model organism *R. philippinarum* from experimental data, which, to our knowledge, has never been reported before. Concerning OTNE, this work provides the first BCF and toxicity data, thereby contributing to the knowledge about this chemical in exposure scenarios. The little bioaccumulation field data for some of the compounds tested makes comparison difficult, taking into account the high seasonal patterns that some UV filters have in the environment. Moreover, it was demonstrated that the PCPs selected could induce changes in phase I and phase II of xenobiotic metabolism, induce antioxidant enzymes, and produce oxidative stress and even DNA damage in *R. philippinarum.*

However, further research should be done applying a non-targeted approach to evaluate biotransformation products of parent compounds since the metabolization of some target chemicals in other aquatic species has been observed in previous works. Additionally, a complementary approach using bioaccumulation data, biomarker responses, and omic techniques could contribute to identifying the main pathways involved during the metabolization of these products in organisms.

This study makes a contribution to the understanding of the bioaccumulation and toxicological effects of PCPs in aquatic organisms and, although more research is needed, it could contribute to support future regulations.

### Supplementary information

Below is the link to the electronic supplementary material.

Supplementary file1 (DOCX 31 KB)

## Data Availability

Not applicable.

## References

[CR1] Aguirre-Martínez GV, Martín-Díaz ML (2020). A multibiomarker approach to assess toxic effects of wastewater treatment plant effluents and activated defence mechanisms in marine (*Ruditapes philippinarum*) and fresh water (*Corbicula fluminea*) bivalve species. Ecotoxicol.

[CR2] Aguirre-Martínez GV, DelValls TA, Martín-Díaz ML (2016). General stress, detoxification pathways, neurotoxicity and genotoxicity evaluated in *Ruditapes*
*philippinarum* exposed to human pharmaceuticals. Ecotoxicol Environ Saf.

[CR3] Aguirre-Martinez GV (2021**)** Effective biomarkers to assess the toxicity of pharmaceutical residues on marine bivalve. *In:* Pharmaceuticals in Marine and Coastal Environments: Occurrence, Effects and Challenges in a Changing World. Estuar Coast Shelf Sci Series, 1st Edition, 1:419–455

[CR4] Arnot JA, Gobas FAPC (2006). A review of bioconcentration factor (BCF) and bioaccumulation factor (BAF) assessments for organic chemicals in aquatic organisms. Environ Rev.

[CR5] Arpin-Pont L, Martínez-Bueno MJ, Gomez E, Fenet H (2016). Occurrence of PPCPs in the marine environment: a review. Environ Sci Pollut Res.

[CR6] Bachelot M, Li Z, Munaron D, Le Gall P, Casellas C, Fenet H, Gomez E (2012). Organic UV filter concentrations in marine mussels from French coastal regions. Sci Total Environ.

[CR7] Bayen S, Segovia Estrada E, Zhang H, Lee WK, Juhel G, Smedes F, Kelly BC (2019). Partitioning and bioaccumulation of legacy and emerging hydrophobic organic chemicals in mangrove ecosystems. Environ Sci Technol.

[CR8] Bedoux G, Roig B, Thomas O, Dupont V, Le Bot B (2012). Occurrence and toxicity of antimicrobial triclosan and by-products in the environment. Environ Sci Pollut Res.

[CR9] Beers RF, Sizer IW (1952). A spectrophotometric method for measuring the breakdown of hydrogen peroxide by catalase. J BBol Chem.

[CR10] Bester K (2009) Analysis of musk fragrances in environmental samples. J Chromatogr A 1216(3):470–480 10.1016/j.chroma.2008.08.093 18786673

[CR11] Beyer J, Green NW, Brooks S, Allan IJ, Ruus A, Gomes T, Bråte ILN, Schøyen M (2017). Blue mussels (Mytilus edulis spp.) as sentinel organisms in coastal pollution monitoring: A review. Mar Environ Res.

[CR12] Biel-Maeso M, Corada-Fernández C, Lara-Martín PA (2019). Removal of personal care products (PCPs) in wastewater and sludge treatment and their occurrence in receiving soils. Water Res.

[CR13] Binelli A, Cogni D, Parolini M, Riva C, Provini A (2009). In vivo experiments for the evaluation of genotoxic and cytotoxic effects of Triclosan in Zebra mussel hemocytes. Aquat Toxicol.

[CR14] Binelli A, Parolini M, Pedriali A, Provini A (2011). Antioxidant activity in the zebra mussel (Dreissena polymorpha) in response to triclosan exposure. Water Air Soil Pollut.

[CR15] Blasco J, Puppo J (1999). Effect of heavy metals (Cu, Cd and Pb) on aspartate and alanine aminotransferase in Ruditapes philippinarum (Mollusca: Bivalvia). Comp. Biochem Physiol - C Pharmacol Toxicol Endocrinol.

[CR16] Blüthgen N, Zucchi S, Fent K (2012). Effects of the UV filter benzophenone-3 (oxybenzone) at low concentrations in zebrafish (Danio rerio). Toxicol Appl Pharmacol.

[CR17] Blüthgen N, Meili N, Chew G, Odermatt A, Fent K (2014). Accumulation and effects of the UV-filter octocrylene in adult and embryonic zebrafish (Danio rerio). Sci Total Environ.

[CR18] Bonnefille B, Arpin-Pont L, Gomez E, Fenet H, Courant F (2017). Metabolic profiling identification of metabolites formed in Mediterranean mussels (Mytilus galloprovincialis) after diclofenac exposure. Sci Total Environ.

[CR19] Bordalo D, Leite C, Almeida Â, Soares AMVM, Pretti C, Freitas R (2020). Impacts of UV filters in Mytilus galloprovincialis: preliminary data on the acute effects induced by environmentally relevant concentrations. Sustain.

[CR20] Bradford MM (1976). A rapid and sensitive method for the quantitation of microgram quantities of protein utilizing the principle of protein-dye binding. Anal Biochem.

[CR21] Cadena-Aizaga MI, Montesdeoca-Esponda S, Sosa-Ferrera Z, Santana-Rodríguez JJ (2022). Occurrence and environmental hazard of organic UV filters in seawater and wastewater from Gran Canaria Island (Canary Islands, Spain). Environ Pollut.

[CR22] Campos D, Gravato C, Quintaneiro C, Golovko O, Žlábek V, Soares AMVM, Pestana JLT (2017). Toxicity of organic UV-filters to the aquatic midge Chironomus riparius. Ecotoxicol Environ Saf.

[CR23] Canesi L, Ciacci C, Lorusso LC, Betti M, Gallo G, Pojana G, Marcomini A (2007). Effects of triclosan on Mytilus galloprovincialis hemocyte function and digestive gland enzyme activities: possible modes of action on non target organisms. Comp Biochem Physiol Part C Toxicol Pharmacol.

[CR24] Chaves Lopes F, de Castro RM, Caldas Barbosa S, Primel EG, de Martinez Gaspar Martins C (2020) Effect of the UV filter, benzophenone-3, on biomarkers of the yellow clam (Amarilladesma mactroides) under different pH conditions. Mar Pollut Bull 158:111401 10.1016/j.marpolbul.2020.111401 32753186

[CR25] Chiriac FL, Lucaciu IE, Paun I, Pirvu F, Gheorghe S (2022) In vivo bioconcentration, distribution and metabolization of benzophenone-3 (BP-3) by Cyprinus carpio (European Carp). Foods 11(11):1627 10.3390/foods11111627 PMC9180567 35681379

[CR26] Cohen MB, Duvel DL (1988). Characterization of the inhibition of glutathione reductase and the recovery of enzyme activity in exponentially growing murine leukemia (11210) cells treated with 1, 3-bis (2-chloroethyl)-1-nitrosourea. Biochem Pharmacol.

[CR27] Contreras-Vergara CA, Harris-Valle C, Sotelo-Mundo RR, Yepiz-Plascencia G (2004). A mu-class glutathione S-transferase from the marine shrimp Litopenaeus vannamei: molecular cloning and active-site structural modeling. J Biochem Mol Toxicol.

[CR28] Coronado M, De Haro H, Deng X, Rempel MA, Lavado R, Schlenk D (2008). Estrogenic activity and reproductive effects of the UV-filter oxybenzone (2-hydroxy-4-methoxyphenyl-methanone) in fish. Aquat Toxicol.

[CR29] Cortez FS, Seabra Pereira CD, Santos AR, Cesar A, Choueri RB, Martini GDA, Bohrer-Morel MB (2012). Biological effects of environmentally relevant concentrations of the pharmaceutical triclosan in the marine mussel Perna perna (Linnaeus, 1758). Environ Pollut.

[CR30] Cuccaro A, Freitas R, De Marchi L, Oliva M, Pretti C (2022a) UV-filters in marine environments: a review of research trends, meta-analysis, and ecotoxicological impacts of 4-methylbenzylidene-camphor and benzophenone-3 on marine invertebrate communities. Environ Sci Pollut Res 29(43):64370–64391 10.1007/s11356-022-21913-4 35852751

[CR31] Cuccaro A, De Marchi L, Oliva M, Battaglia F, Meucci V, Fumagalli G, Freitas R, Pretti C (2022b) Ecotoxicological effects of the UV-filter 4-MBC on sperms and adults of the mussel Mytilus galloprovincialis. Environ Res 213:113739 10.1016/j.envres.2022.113739 35750122

[CR32] Cunha SC, Trabalón L, Jacobs S, Castro M, Fernandez-Tejedor M, Granby K, Verbeke W, Kwadijk C, Ferrari F, Robbens J, Sioen I, Pocurull E, Marques A, Fernandes JO, Domingo JL (2018). UV-filters and musk fragrances in seafood commercialized in Europe Union: Occurrence, risk and exposure assessment. Environ Res.

[CR33] Cuquerella MC, Lhiaubet-vallet V, Cadet J, Miranda MA (2012). Benzophenone photosensitized DNA damage. Acc Chem Res.

[CR34] Dagnino A, Allen JI, Moore MN, Broeg K, Canesi L, Viarengo A (2007). Development of an expert system for the integration of biomarker responses in mussels into an animal health index. Biomarkers.

[CR35] Dann AB, Hontela A (2011). Triclosan: environmental exposure, toxicity and mechanisms of action. J Appl Toxicol.

[CR36] de García SO, García-Encina PA, Irusta-Mata R (2017). The potential ecotoxicological impact of pharmaceutical and personal care products on humans and freshwater, based on USEtox™ characterization factors. A Spanish case study of toxicity impact scores. Sci Total Environ.

[CR37] De Lafontaine Y, Gagné F, Blaise C, Costan G, Gagnon P, Chan HM (2000). Biomarkers in zebra mussels (Dreissena polymorpha) for the assessment and monitoring of water quality of the St Lawrence River (Canada). Aquat Toxicol.

[CR38] De Marchi L, Freitas R, Oliva M, Cuccaro A, Manzini C, Tardelli F, Andrade M, Costa M, Leite C, Morelli A, Chiellini F, Pretti C (2020). Does salinity variation increase synergistic effects of triclosan and carbon nanotubes on Mytilus galloprovincialis? Responses on adult tissues and sperms. Sci Total Environ.

[CR39] Dhillon GS, Kaur S, Pulicharla R, Brar SK, Cledón M, Verma M, Surampalli RY (2015). Triclosan: current status, occurrence, environmental risks and bioaccumulation potential. Int J Environ Res Public Health.

[CR40] Dodder NG, Maruya KA, Lee Ferguson P, Grace R, Klosterhaus S, La Guardia MJ, Lauenstein GG, Ramirez J (2014). Occurrence of contaminants of emerging concern in mussels (Mytilus spp.) along the California coast and the influence of land use, storm water discharge, and treated wastewater effluent. Mar Pollut Bull.

[CR41] Donkin P, Widdows J, Evans SV, Brinsley MD (1991). QSARs for the sublethal responses of marine mussels (Mytilus edulis). Sci Total Environ.

[CR42] Ebele AJ, Abdallah M-E, Harrad S (2017). Pharmaceuticals and personal care products (PPCPs) in the freshwater aquatic environment. Emerging Contaminants.

[CR43] ECHA (2023) Available at: https://echa.europa.eu/de/brief-profile/-/briefprofile/100.025.683. Accessed 20 Dec 2023

[CR44] Ehiguese FO, Alam MR, Pintado-Herrera MG, Araújo CVM, Martin-Diaz ML (2020) Potential of environmental concentrations of the musks galaxolide and tonalide to induce oxidative stress and genotoxicity in the marine environment. Mar Environ Res 160:105019 10.1016/j.marenvres.2020.105019 32907733

[CR45] Escarrone ALV, Caldas SS, Primel EG, Martins SE, Nery LEM (2016). Uptake, tissue distribution and depuration of triclosan in the guppy Poecilia vivipara acclimated to freshwater. Sci Total Environ.

[CR46] European Commission (2003) Technical Guidance Document (TGD) on risk assessment in support of Commission Directive 93/67/EEC on risk assessment for new notified substances and Commission Regulation (EC) No 1488/94 on risk assessment for existing substances and Directive 98/8/EC of the European parliament and of the council concerning the placing of biocidal products on the market. The European Community, Brussels, Belgium, 2003

[CR47] Fair PA, Lee H-B, Adams J, Darling C, Pacepavicius G, Alaee M, Bossart GD, Henry N, Muir D (2009). Occurrence of triclosan in plasma of wild Atlantic bottlenose dolphins (Tursiops truncatus) and in their environment. Environ Pollut.

[CR48] Falfushynska H, Sokolov EP, Fisch K, Gazie H, Schulz-Bull DE, Sokolova IM (2021). Biomarker-based assessment of sublethal toxicity of organic UV filters (ensulizole and octocrylene) in a sentinel marine bivalve Mytilus edulis. Sci Total Environ.

[CR49] FAO (2013) P. Goulletquer (Ed.), Cultured aquatic species information programme. Ruditapes Philippinarum, FAO Fisheries and Aquaculture Department, Rome (2013) www.fao.org/fishery/culturedspecies/Ruditapes_philippinarum/en. Accessed 20 Dec 2023

[CR50] Fenni F, Sunyer-Caldú A, Ben Mansour H, Diaz-Cruz MS (2022). Contaminants of emerging concern in marine areas: first evidence of UV filters and paraben preservatives in seawater and sediment on the eastern coast of Tunisia. Environ Pollut.

[CR51] Flohé L, Günzler WA (1984). [12] Assays of glutathione peroxidase. In Methods in Enzymology Acad Press.

[CR52] Freitas R, Coppola F, Costa S, Pretti C, Intorre L, Meucci V, Soares AMVM, Solé M (2019). The influence of temperature on the effects induced by Triclosan and Diclofenac in mussels. Sci Total Environ.

[CR53] Gagné F, Blaise C (1993). Hepatic metallothionein level and mixed function oxidase activity in fingerling rainbow trout (Oncorhynchus mykiss) after acute exposure to pulp and paper mill effluents. Water Res.

[CR54] Gagné F, Blaise C (1995). Evaluation of the genotoxicity of environmental contaminants in sediments to rainbow trout hepatocytes. Environ Toxicol Wat Qual.

[CR55] Gago-Ferrero P, Alonso MB, Bertozzi CP, Marigo J, Barbosa L, Cremer M, Secchi ER, Azevedo A, Lailson-Brito J, Torres JPM, Malm O, Eljarrat E, Díaz-Cruz MS, Barceló D (2013). First determination of UV filters in marine mammals. Octocrylene Levels in Franciscana Dolphins. Environ Sci Technol.

[CR56] Garcia-Galan MJ, Sordet M, Buleté A, Garric J, Vulliet E (2017). Evaluation of the influence of surfactants in the bioaccumulation kinetics of sulfamethoxazole and oxazepam in benthic invertebrates. Sci Total Environ.

[CR57] Gatidou G, Vassalou E, Thomaidis NS (2010). Bioconcentration of selected endocrine disrupting compounds in the Mediterranean mussel, Mytilus galloprovincialis. Mar Pollut Bull.

[CR58] Giraldo A, Montes R, Rodil R, Quintana JB, Vidal-Liñán L, Beiras R (2017). Ecotoxicological evaluation of the UV filters ethylhexyl dimethyl p-aminobenzoic acid and octocrylene using marine organisms Isochrysis galbana, Mytilus galloprovincialis and Paracentrotus lividus. Arch Environ Contam Toxicol.

[CR59] Gooding MP, Newton TJ, Bartsch MR, Hornbuckle KC (2006). Toxicity of synthetic musks to early life stages of the freshwater mussel Lampsilis cardium. Arch Environ Contam Toxicol.

[CR60] Grabicova K, Fedorova G, Burkina V, Steinbach C, Schmidt-Posthaus H, Zlabek V, Kocour Kroupova H, Grabic R, Randak T (2013). Presence of UV filters in surface water and the effects of phenylbenzimidazole sulfonic acid on rainbow trout (Oncorhynchus mykiss) following a chronic toxicity test. Ecotoxicol Environ Saf.

[CR61] Gupta DK, Palma JM, Corpas FJ (2016) Redox state as a central regulator of plant-cell stress responses. Camb Int Law J

[CR62] Hartman JH, Widmayer SJ, Bergemann CM, King DE, Morton KS, Romersi RF, Jameson LE, Leung MCK, Andersen EC, Taubert S, Meyer JN (2021). Xenobiotic metabolism and transport in Caenorhabditis elegans. J. Toxicol. Environ Heal - Part B Crit Rev.

[CR63] Impellitteri F, Multisanti CR, Rusanova P, Piccione G, Falco F, Faggio C (2023). Exploring the impact of contaminants of emerging concern on fish and invertebrates physiology in the Mediterranean Sea. Biology.

[CR64] Ismail SN, Müller EC, Morgan RR, Luthy GR (2014). Uptake of contaminants of emerging concern by the bivalves Anodonta californiensis and Corbicula fluminea. Environ Sci Technol.

[CR65] Janero DR (1990) Malondialdehyde and thiobarbituric acid-reactivity as diagnostic indices of lipid peroxidation and peroxidative tissue injury. Free Radic Biol Med 9(6):515–540 10.1016/0891-5849(90)90131-2 2079232

[CR66] Kim S, Choi K (2014). Occurrences, toxicities, and ecological risks of benzophenone-3, a common component of organic sunscreen products: a mini-review. Environ Int.

[CR67] Kim JW, Ishibashi H, Yamauchi R, Ichikawa N, Takao Y, Hirano M, Arizono K (2009). Acute toxicity of pharmaceutical and personal care products on freshwater crustacean (*Thamnocephalus platyurus*) and fish (*Oryzias latipes*). J Toxicol Sci.

[CR68] Kinnberg KL, Petersen GI, Albrektsen M, Minghlani M, Awad SM, Holbech BF, Green JW, Bjerregaard P, Holbech H (2015). Endocrine-disrupting effect of the ultraviolet filter benzophenone-3 in zebrafish, Danio rerio. Environ Toxicol Chem.

[CR69] Klaschka U, von der Ohe PC, Bschorer A, Krezmer S, Sengl M, Letzel M (2013). Occurrences and potential risks of 16 fragrances in five German sewage treatment plants and their receiving waters. Environ Sci Pollut Res.

[CR70] Kookana RS, Shareef A, Fernandes MB, Hoare S, Gaylard S, Kumar A (2013). Bioconcentration of triclosan and methyl-triclosan in marine mussels (Mytilus galloprovincialis) under laboratory conditions and in metropolitan waters of Gulf St Vincent, South Australia. Mar Pollut Bull.

[CR71] Kunz PY, Gries T, Fent K (2006). The ultraviolet filter 3-benzylidene camphor adversely affects reproduction in fathead minnow (pimephales promelas). Toxicol Sci.

[CR72] Labille J, Slomberg D, Catalano R, Robert S, Apers-Tremelo ML, Boudenne JL, Manasfi T, Radakovitch O (2020). Assessing UV filter inputs into beach waters during recreational activity: a field study of three French Mediterranean beaches from consumer survey to water analysis. Sci Total Environ.

[CR73] Lara-Martín PA, Renfro AA, Cochran JK, Brownawell BJ (2015). Geochronologies of pharmaceuticals in a sewage-impacted estuarine urban setting (Jamaica Bay, New York). Environ Sci Technol.

[CR74] Li Y, Schellhorn HE (2007). New developments and novel therapeutic perspectives for vitamin C. J Nutr.

[CR75] Liao H, Yang Z, Dou Z, Sun F, Kou S, Zhang Z, Huang X, Bao Z (2019). Impact of ocean acidification on the energy metabolism and antioxidant responses of the Yesso scallop (Patinopecten yessoensis). Front Physiol.

[CR76] Lietti E, Marin MG, Matozzo V, Polesello S, Valsecchi S (2007). Uptake and elimination of 4-nonylphenol by the clam Tapes philippinarum. Arch Environ Contam Toxicol.

[CR77] Mackay D (1982). Correlation of bioconcentration factors. Environ Sci Technol.

[CR78] Matozzo V, Costa Devoti A, Marin MG (2012). Immunotoxic effects of triclosan in the clam Ruditapes philippinarum. Ecotoxicol.

[CR79] Matozzo V, Formenti A, Donadello G, Marin MG (2012). A multi-biomarker approach to assess effects of triclosan in the clam Ruditapes philippinarum. Mar Environ Res.

[CR80] Maynou F, Costa S, Freitas R, Solé M (2021). Effects of triclosan exposure on the energy budget of Ruditapes philippinarum and R. decussatus under climate change scenarios. Sci Total Environ.

[CR81] McDonough K, Casteel K, Zoller A, Wehmeyer K, Hulzebos E, Rila JP, Salvito D, Federle T (2017). Probabilistic determination of the ecological risk from OTNE in aquatic and terrestrial compartments based on US-wide monitoring data. Chemosphere.

[CR82] McFarland VA, Inouye LS, Lutz CH, Jarvis AS, Clarke JU, McCant DD (1999). Biomarkers of oxidative stress and genotoxicity in livers of field- collected brown bullhead, Ameiurus nebulosus. Arch Environ Contam Toxicol.

[CR83] Meylan WM, Howard PH, Boethling RS, Aronson D, Printup H, Gouchie S (1999). Improved method for estimating bioconcentration/bioaccumulation factor from octanol/water partition coefficient. Environ Toxicol Chem.

[CR84] Moschino V, Delaney E, Da Ros L (2012). Assessing the significance of Ruditapes philippinarum as a sentinel for sediment pollution: bioaccumulation and biomarker responses. Environ Pollut.

[CR85] Nakajima D, Asada S, Kageyama S, Yamamoto T, Kuramochi H, Tanaka N, Takeda K, Goto S (2006). Activity related to the carcinogenicity of plastic additives in the benzophenone group. J UOEH.

[CR86] Nipen M, Vogt RD, Bohlin-Nizzetto P, Borgå K, Mwakalapa EB, Borgen AR, Schlabach M, Christensen G, Mmochi AJ, Breivik K (2022). Increasing trends of legacy and emerging organic contaminants in a dated sediment core from East-Africa. Front Environ Sci.

[CR87] O’Donovan S, Mestre NC, Abel S, Fonseca TG, Carteny CC, Willems T, Prinsen E, Cormier B, Keiter SS, Bebianno MJ (2020). Effects of the UV filter, oxybenzone, adsorbed to microplastics in the clam Scrobicularia plana. Sci Total Environ.

[CR88] Olive P, Chan APS, Cu CS (1988). Comparison between the DNA precipitation and alkali unwinding assays for detecting DNA strand breaks and cross-links. Cancer Res.

[CR89] Ozaki N, Tanaka T, Kindaichi T, Ohashi A (2021) Photodegradation of fragrance materials and triclosan in water: direct photolysis and photosensitized degradation. Environ Technol Innov 23

[CR90] Paredes E, Perez S, Rodil R, Quintana JB, Beiras R (2014). Ecotoxicological evaluation of four UV filters using marine organisms from different trophic levels Isochrysis galbana, Mytilus galloprovincialis, Paracentrotus lividus, and Siriella armata. Chemosphere.

[CR91] Parolini M, Pedriali A, Binelli A (2013). Application of a biomarker response index for ranking the toxicity of five pharmaceutical and personal care products (PPCPs) to the bivalve Dreissena polymorpha. Arch Environ Contam Toxicol.

[CR92] Pawlowski S, Lanzinger AC, Dolich T, Füßl S, Salinas ER, Zok S, Weiss B, Hefner N, Van Sloun P, Hombeck H, Klingelmann E, Petersen-Thiery M (2019). Evaluation of the bioaccumulation of octocrylene after dietary and aqueous exposure. Sci Total Environ.

[CR93] Pemberthy MD, Padilla Y, Echeverri A, Peñuela GA (2020). Monitoring pharmaceuticals and personal care products in water and fish from the Gulf of Urabá, Colombia. Heliyon.

[CR94] Pico Y, Belenguer V, Corcellas C, Diaz-Cruz MS, Eljarrat E, Farré M, Gago-Ferrero P, Huerta B, Navarro-Ortega A, Petrovic M, Rodríguez-Mozaz S, Sabater L, Santín G, Barcelo D (2019). Contaminants of emerging concern in freshwater fish from four Spanish Rivers. Sci Total Environ.

[CR95] Pintado-Herrera MG, González-Mazo E, Lara-Martín PA (2014). Atmospheric pressure gas chromatography-time-of-flight-mass spectrometry (APGC-ToF-MS) for the determination of regulated and emerging contaminants in aqueous samples after stir bar sorptive extraction (SBSE). Anal Chim Acta.

[CR96] Pintado-Herrera MG, González-Mazo E, Lara-Martín PA (2016). In-cell clean-up pressurized liquid extraction and gas chromatography–tandem mass spectrometry determination of hydrophobic persistent and emerging organic pollutants in coastal sediments. J Chromatogr A.

[CR97] Pintado-Herrera MG, Allan IJ, González-Mazo E, Lara-Martín PA (2020). Passive samplers vs sentinel organisms: one-year monitoring of priority and emerging contaminants in coastal waters. Environ Sci Technol.

[CR98] Pirone G, Coppola F, Pretti C, Soares AMVM, Solé M, Freitas R (2019). The effect of temperature on triclosan and lead exposed mussels. Comp. Biochem. Physiol Part - B Biochem Mol Biol.

[CR99] Regnault C, Willison J, Veyrenc S, Airieau A, Méresse P, Fortier M, Fournier M, Brousseau P, Raveton M, Reynaud S (2016). Metabolic and immune impairments induced by the endocrine disruptors benzo[a]pyrene and triclosan in Xenopus tropicalis. Chemosphere.

[CR100] Reiner JL, Kannan K (2011). Polycyclic musks in water, sediment, and fishes from the upper Hudson River, New York, USA. Water Air Soil Pollut.

[CR101] Riva C, Cristoni S, Binelli A (2012). Effects of triclosan in the freshwater mussel Dreissena polymorpha: a proteomic investigation. Aquat Toxicol.

[CR102] Rodríguez-Fuentes G, Sandoval-Gío JJ, Arroyo-Silva A, Noreña-Barroso E, Escalante-Herrera KS, Olvera-Espinosa F (2015). Evaluation of the estrogenic and oxidative stress effects of the UV filter 3-benzophenone in zebrafish (Danio rerio) eleuthero-embryos. Ecotoxicol Environ Saf.

[CR103] Rolton A, Champeau O, Barrick A, Boundy M, Tremblay LA, Vignier J (2022). Characterization of the effects of triclosan on sperm and embryos of Mytilus and Perna mussel species. Aquat Toxicol.

[CR104] Sánchez-Quiles D, Tovar-Sánchez A (2015). Are sunscreens a new environmental risk associated with coastal tourism?. Environ Int.

[CR105] Santonocito M, Salerno B, Trombini C, Tonini F, Pintado-Herrera MG, Martínez-Rodríguez G, Blasco J, Lara-Martín PA, Hampel M (2020) Stress under the sun: effects of exposure to low concentrations of UV-filter 4- methylbenzylidene camphor (4-MBC) in a marine bivalve filter feeder, the Manila clam Ruditapes philippinarum. Aquat Toxicol 221:105418 10.1016/j.aquatox.2020.105418 32078887

[CR106] Saunders LJ, Hoffman AD, Nichols JW, Gobas FAPC (2020). Dietary bioaccumulation and biotransformation of hydrophobic organic sunscreen agents in rainbow trout. Environ Toxicol Chem.

[CR107] Sauvé S, Desrosiers M (2014). A review of what is an emerging contaminant. Chem Cent J.

[CR108] Schnitzler JG, Frédérich B, Dussenne M, Klaren PHM, Silvestre F, Das K (2016). Triclosan exposure results in alterations of thyroid hormone status and retarded early development and metamorphosis in Cyprinodon variegatus. Aquat Toxicol.

[CR109] Sendra M, Pintado-Herrera MG, Aguirre-Martínez GV, Moreno-Garrido I, Martin-Díaz LM, Lara-Martín PA, Blasco J (2017) Are the TiO2 NPs a “Trojan horse” for personal care products (PCPs) in the clam Ruditapes philippinarum?. Chemosphere 185:192–204 10.1016/j.chemosphere.2017.07.009 28697425

[CR110] Seoane M, Cid Á, Herrero C, Esperanza M (2021). Comparative acute toxicity of benzophenone derivatives and bisphenol analogues in the Asian clam Corbicula fluminea. Ecotoxicology.

[CR111] Snell K, Mullock B (1987) Evaluation of lipid peroxidation in lipids and biological membranes. Biochem Toxicol A Pract Approach, IRL Press, Oxford, 127–152

[CR112] Stien D, Clergeaud F, Rodrigues AMS, Lebaron K, Pillot R, Romans P, Fagervold S, Lebaron P (2019). Metabolomics reveal that octocrylene accumulates in pocillopora damicornis tissues as fatty acid conjugates and triggers coral cell mitochondrial dysfunction. Anal Chem.

[CR113] Stien D, Suzuki M, Rodrigues AMS, Yvin M, Clergeaud F, Thorel E, Lebaron P (2020). A unique approach to monitor stress in coral exposed to emerging pollutants. Sci Rep.

[CR114] Tamura I, Kagota KI, Yasuda Y, Yoneda S, Morita J, Nakada N, Kameda Y, Kimura K, Tatarazako N, Yamamoto H (2013). Ecotoxicity and screening level ecotoxicological risk assessment of five antimicrobial agents: triclosan, triclocarban, resorcinol, phenoxyethanol and p-thymol. J Appl Toxicol.

[CR115] Tato T, Salgueiro-González N, León VM, González S, Beiras R (2018). Ecotoxicological evaluation of the risk posed by bisphenol A, triclosan, and 4-nonylphenol in coastal waters using early life stages of marine organisms (Isochrysis galbana, Mytilus galloprovincialis, Paracentrotus lividus, and Acartia clausi). Environ Pollut.

[CR116] Tsui MMP, Chen L, He T, Wang Q, Hu C, Lam JCW, Lam PKS (2019). Organic ultraviolet (UV)filters in the South China sea coastal region: environmental occurrence, toxicological effects and risk assessment. Ecotoxicol Environ Saf.

[CR117] Vidal-Liñán L, Villaverde-de-Sáa E, Rodil R, Quintana JB, Beiras R (2018). Bioaccumulation of UV filters in Mytilus galloprovincialis mussel. Chemosphere.

[CR118] Wang F, Liu F, Chen W, Xu R, Wang W (2018). Effects of triclosan (TCS) on hormonal balance and genes of hypothalamus-pituitary- gonad axis of juvenile male Yellow River carp (Cyprinus carpio). Chemosphere.

[CR119] Weatherly LM, Gosse JA (2017) Triclosan exposure, transformation, and human health effects. J Toxicol Environ Health B Crit Rev 20(8):447–469 10.1080/10937404.2017.1399306 PMC6126357 29182464

[CR120] Webb S, Gaw S, Marsden ID, McRae NK (2020). Biomarker responses in New Zealand green-lipped mussels Perna canaliculus exposed to microplastics and triclosan. Ecotoxicol Environ Saf.

[CR121] Wilson BA, Smith VH, deNoyelles F, Larive CK (2003). Effects of three pharmaceutical and personal care products on natural freshwater algal assemblages. Environ Sci Technol.

[CR122] Ziarrusta H, Mijangos L, Montes R, Rodil R, Anakabe E, Izagirre U, Prieto A, Etxebarria N, Olivares M, Zuloaga O (2018). Study of bioconcentration of oxybenzone in gilt-head bream and characterization of its by-products. Chemosphere.

[CR123] Zicarelli G, Multisanti CR, Falco F, Faggio C (2022). Evaluation of toxicity of personal care products (PCPs) in freshwaters: zebrafish as a model. Environ Toxicol Pharmacol.

