# Supplementary material for: Personal care products: an emerging threat to the marine bivalve Ruditapes philippinarum
Source: Environ Sci Pollut Res Int. 2024 Feb 20;31(13):20461–76. doi: 10.1007/s11356-024-32391-1 (PMC10927873; doi:10.1007/s11356-024-32391-1)
Supplement: Supplementary file 1 — Supplementary file1 (DOCX 31 KB) [file 11356_2024_32391_MOESM1_ESM.docx]

**Supplementary Information**

**Table S1.** Chemical properties

| Compound | Molecular weight | *Water solubility (mg/L) | *logK_ow_ | Affinity to lipid tissues |
| --- | --- | --- | --- | --- |
| Triclosan (antibacterial) | 289.54 | 4.621 | 4.76 | High |
| OTNE  (fragrance) | 234.37 | 1.077 | 5.18 | Very high |
| BP3  (UV filter) | 228.24 | 68.56 | 3.8 | High |
| OC  (UV filter) | 361.47 | 0.0038 | 6.8 | Very high |

*www.chemspider.com

**Table S2.** Experimental conditions.

| Number of tanks | 20 |
| --- | --- |
| Number of bivalves per tank | 50 |
| Volume of water in tanks (L) | 40 |
| Water renewal (h) | 48 |
| Sampling points (days) | Uptake period: 2, 7, 14, 26  Depuration period: 3, 7 |
| Number of individuals at each sampling point | Bioconcentration´s analysis: 4  Biomarker´s analysis: 3 |

**Table S3.** QSAR equations obtained from the literature.

| Reference | | Equation | Observations |
| --- | --- | --- | --- |
| Mackay, 1982 | 1 | Log BCF = log K_ow_ – 1.32 | Estimated on 63 compounds. |
| Arnot and Gobas, 2006 | 2 | Log BCF = 0.92 · log K_ow_ – 1.45 | Based on a review of 5317 BCF published in 392 scientific literature. |
| Donkin et al., 1991 | 3 | Log BCF = 1.03 · log K_ow_ – 2.11 |  |
| European Technical Guidance (2003) | 4a  4b | Log BCF = 0.85 · log K_ow_ – 0.70  __________________________  Log BCF = -0.20 · (log K_ow_)^2^ + 2.74 · log K_ow_ – 4.72 | Estimated on 50 compounds.  __________________________  For compounds with log K_ow_ > 6 |

**Table S4**. Mean recovery of the extraction for the target analytes in clams (±standard deviations, n=3).

| Compounds | Recovery (%) |
| --- | --- |
| Triclosan (TCS) | 108± 3 |
| OTNE | 85±11 |
| Benzophenone 3(BP3) | 97±9 |
| Octocrylene (OC) | 80±14 |

**Table S5.** Concentrations of the target compounds measured in spiked seawater tanks with clams (mean, µg L^-1^) and losses (%).

| Time after spiking (h) | OTNE | BP3 | OC | TCS |
| --- | --- | --- | --- | --- |
| 2 | 3.8 | 9.5 | 7.7 | 5.4 |
| 5 | 0.7 | 8.1 | 2.1 | 1.7 |
| 24 | 0.3 | 5.8 | 0.7 | 0.25 |
| 48 | 0.2 | 3.2 | 0.3 | 0.3 |
| Loss (%) | 95 | 66 | 96 | 94 |

**Table S6.** Concentrations of the chemicals measured in control and solvent control clams (Manila clam) (ng/g dw ± sd, n=8).

|  | OTNE | BP3 | OC | TCS |
| --- | --- | --- | --- | --- |
|  | 43.4 ± 10.5 | 39.5 ± 27.8 | 23.8 ± 0.9 | 26.0 ± 5.7 |

**Table S7.** Concentrations of the chemicals measured in exposed clams (Manila clam) for 26 days and during the depuration period for 10 days (day 3 and day 7) (mean ± standard deviations, n=4, ng/g dw).

| Treatment | OTNE | BP3 | OC | TCS |
| --- | --- | --- | --- | --- |
| Day 0 | 35.7 ± 5.8 | 34.5 ± 12.4 | 33.7 ± 10.1 | 30.3 ± 6.3 |
| Uptake Period | | | | |
| Day 2 | 334.4 ± 45.0 | 733.2 ± 138.5 | 398.0 ± 74.0 | 540.0 ± 170.7 |
| Day 7 | 483.0 ± 70.6 | 9157.4 ± 1487 | 682.2 ± 150 | 863.9 ± 116.4 |
| Day 14 | 617.3 ± 37.8 | 10221.5 ± 680.9 | 766.1 ± 146.0 | 1699.1 ± 222.4 |
| Day 26 | 682.0 ± 175.6 | 24058.8 ± 3057.4 | 806.1 ± 124.8 | 1523.2 ± 284.6 |
| Depuration period | | | | |
| Day 3 | 271.8 ± 79.4 | 3519.2 ± 1260.9 | 390.5 ± 71.5 | 875.9 ± 59.5 |
| Day 7 | 140.6 ± 23.3 | 841.3 ± 59.6 | 211.4 ± 58.5 | - 1. ± 91.4 |

**Table S8.** Kinetic parameters and log BCF experimental and calculated from QSAR equations.

| Kinetic Parameters | OTNE | BP3 | OC | TCS |
| --- | --- | --- | --- | --- |
| k*_uptake_*  (L kg^-1^ day^-1^) | 357.5 | 435.5 | 467.3 | 196.8 |
| K*_depur_*  (day^-1^) | 0.225 | 0.416 | 0.212 | 0.231 |
| BCF  (L kg^-1^) | 1589 | 1047 | 2204 | 852 |
| *t_1/2_*  (day) | 3.1 | 1.6 | 3.2 | 3 |
| **QSAR equations (log BCF)** | | | | |
| ***Eq. 1*** | 3.9 | 2.5 | 5.5 | 3.4 |
| ***Eq 2.*** | 3.3 | 2.0 | 4.8 | 2.9 |
| ***Eq. 3*** | 3.2 | 1.8 | 4.9 | 2.8 |
| ***Eq 4a / 4b*** | 3.7 | 2.5 | 4.7 | 3.3 |
| ***This study*** | 3.2 | 3.0 | 3.3 | 2.9 |

**References**

Mackay, D., 1982. Correlation of Bioconcentration Factors. Environ. Sci. Technol. 16, 274–278.

Arnot, J.A., Gobas, F.A.P.C., 2006. A review of bioconcentration factor (BCF) and bioaccumulation factor (BAF) assessments for organic chemicals in aquatic organisms. Environ. Rev. 14, 257–297.

Donkin, P., Widdows, J., Evans, S. V, Brinsley, M.D., 1991. QSARs for the sublethal responses of marine mussels (Mytilus edulis). Sci. Total Environ. 109–110, 461–476.

European Commission, 2003. Technical Guidance Document (TGD) on risk assessment in support of Commission Directive 93/67/EEC on risk assessment for new notified substances and Commission Regulation (EC) No 1488/94 on risk assessment for existing substances and Directive 98/8/EC of the European parliament and of the council concerning the placing of biocidal products on the market. The European Community, Brussels, Belgium., 2003.
